# Mcl-1 Is a Key Regulator of Apoptosis Resistance in *Chlamydia trachomatis*-Infected Cells

**DOI:** 10.1371/journal.pone.0003102

**Published:** 2008-09-01

**Authors:** Krishnaraj Rajalingam, Manu Sharma, Christine Lohmann, Monique Oswald, Oliver Thieck, Christopher J. Froelich, Thomas Rudel

**Affiliations:** 1 Department of Molecular Biology, Max Planck Institute for Infection Biology, Berlin, Germany; 2 Evanston Northwestern Research Institute, Evanston, Illinois, United States of America; Duke University Medical Center, United States of America

## Abstract

*Chlamydia* are obligate intracellular bacteria that cause variety of human diseases. Host cells infected with *Chlamydia* are protected against many different apoptotic stimuli. The induction of apoptosis resistance is thought to be an important immune escape mechanism allowing *Chlamydia* to replicate inside the host cell. Infection with *C. trachomatis* activates the Raf/MEK/ERK pathway and the PI3K/AKT pathway. Here we show that inhibition of these two pathways by chemical inhibitors sensitized *C. trachomatis* infected cells to granzyme B-mediated cell death. Infection leads to the Raf/MEK/ERK-mediated up-regulation and PI3K-dependent stabilization of the anti-apoptotic Bcl-2 family member Mcl-1. Consistently, interfering with Mcl-1 up-regulation sensitized infected cells for apoptosis induced via the TNF receptor, DNA damage, granzyme B and stress. Our data suggest that Mcl-1 up-regulation is primarily required to maintain apoptosis resistance in *C. trachomatis*-infected cells.

## Introduction

*Chlamydia* are obligate-intracellular gram-negative bacteria with an innate biphasic life cycle. The infection starts with the uptake of the metabolically inactive elementary bodies (EBs) by the eukaryotic cell. EBs differentiate to metabolically active reticulate bodies (RBs) which replicate in a vacuole inside the host cell. RBs re-differentiate to EBs, which are then released from the cells to initiate a new cycle of infection. Despite the fact that they are strictly dependent on host eukaryotic cells for their growth, infections with *Chlamydia* are the cause of several human diseases. Among these, *C. pneumoniae* infection induces respiratory disorders [1], whereas *C. trachomatis* has been demonstrated to be the major causative of bacterial sexually transmitted diseases and ocular infections leading to blindness [2].

Modulation of host cell apoptosis is an important immune escape mechanism employed by a broad range of viral, bacterial and parasitic pathogens. For instance, several pathogenic bacteria like *Salmonella* spp. *Shigella* spp. and *Yersinia* spp. induce apoptosis in macrophages to avoid their destruction by these powerful immune effector cells [3]. Obligate intracellular bacteria like *Rickettsia* and *Chlamydia* have evolved strategies to increase the resistance of their host cells for apoptotic stimuli [4]–[7]. Inhibition of host cell apoptosis may protect the replicating bacteria from the action of cytotoxic T cells, which eliminate infected cells by the induction of apoptosis. Moreover, an important aspect of preventing apoptosis in infected cells is the chronic infection. *Chlamydia* spp. can persist inside the infected cells and apoptosis inhibition may even prolong the life span of the host cells [8].

The mechanisms of apoptosis induction have been worked out in great detail. Apoptosis is primarily induced by two major pathways namely the ‘extrinsic’ or the death receptor-mediated or by the ‘intrinsic’ or the mitochondria-mediated pathways. Caspases, the effector proteases are activated either by the binding of death ligand to the receptors or by the release of pro-apoptotic factors from the mitochondria [9]. Initiator caspases are activated independent of cleavage by recruitment to large signaling complexes assembled by the ligation of death receptors. For instance, caspase-8 is activated by the Fas receptor associated *death inducing signaling complex* (DISC) [10] and caspase-9 is activated by the apoptosome triggered by cytochrome c released from mitochondria into the cytosol [11]. Inhibitor of apoptosis proteins (IAPs) constitute an important class of apoptosis regulators as they can directly bind and prevent the activation of effector caspases [12]. During apoptosis, the mitochondrial outer membrane is permeabilized and is primarily accomplished by the activation of “pro-apoptotic” Bcl-2 family members Bax and Bak. Activation of Bax and Bak can be counteracted by the anti-apoptotic Bcl-2 family members like Bcl-2, Bcl-X_L_, Mcl-1 and A1. Mcl-1 was identified as an early induction gene during myeloblastic cell differentiation and has also been established to play a crucial role in the survival and homeostasis of lymphocytes [13], [14]. Mcl-1 has a fast turnover rate and several growth factors modulate the expression of Mcl-1 both at the transcriptional as well as post-translational levels [15]. Mcl-1 has a strong binding affinity for BH3-only family member Bim, and is localized in a complex with Bim and Bak at the mitochondrial outer membrane. The Bim-Mcl-1 complex has been shown to be disrupted during induction of apoptosis [16].

One of the major mechanisms by which the immune system clears intracellular infections is by Cytotoxic T lymphocyte (CTL)-mediated cytotoxicity. CTLs and Natural killer cells (NK) utilize two main pathways to activate target cell death, Fas and Granzyme/perforin. While the FAS-mediated apoptosis pathway also plays an important role in lymphocyte homeostasis, granule-mediated killing is vital for clearing intracellular infection, tumor surveillance and transplant rejection [17]. Granzyme B (GrB), one of the important and well-studied proteases of CTLs is a serine protease with an unusual specificity to cleave substrates at aspartic residues [18]. GrB is stored in cytoplasmic granules in the CTLs and NK cells and is delivered to the target cells in a perforin-dependent manner. Though GrB can directly cleave caspase-3, it is still dependent on the permeabilisation of mitochondrial outer membrane as release of Smac/DIABLO is required to inhibit XIAP [19]. Previous studies have revealed that cells infected with *Chlamydia* resist cytochrome *c* release in response to several apoptotic stimuli [5]–[7]. Consistently, activation of pro-apoptotic Bcl-2 family members Bax and Bak is blocked in infected cells [20], [21]. It has been suggested that *C. trachomatis*-induced apoptosis resistance is dependent on the specific degradation of the BH3-only family members by an uncharacterized chlamydial protease [21]–[23] by the recruitment of BAD and 14-3-3 proteins to the chlamydial inclusion [24] and by the stabilization of IAP-IAP complexes [25].

This study stems from our initial search for the bacteria modulated host effectors responsible for resistance to stress- and GrB-mediated cell death. Contradictory to the published observations, we have failed to detect a specific degradation of the BH3 only family members in the *C. trachomatis* infected cells. Using epithelial cells as infection model, we demonstrate here that *C. trachomatis* infection activates both the Raf/MEK/ERK pathway as well as the PI3K/AKT pathway to resist apoptosis induced by GrB, stress and death receptor. Infection with *C. trachomatis* leads to the MEK-dependent up-regulation of Mcl-1 mRNA and PI3K-dependent stabilization of Mcl-1 protein levels. Depletion of Mcl-1 reverses the block in mitochondrial outer membrane permeabilization and sensitizes infected cells to apoptosis induced by GrB, stress and death receptor-mediated pathways. Our data suggest the up-regulation and stabilization of Mcl-1 are crucial events for the apoptosis resistance of *C. trachomatis*-infected cells for a broad range of apoptotic stimuli.

## Results

### Mcl-1 is up-regulated in a MAPK-dependent fashion in infected cells

**Figure 1:**
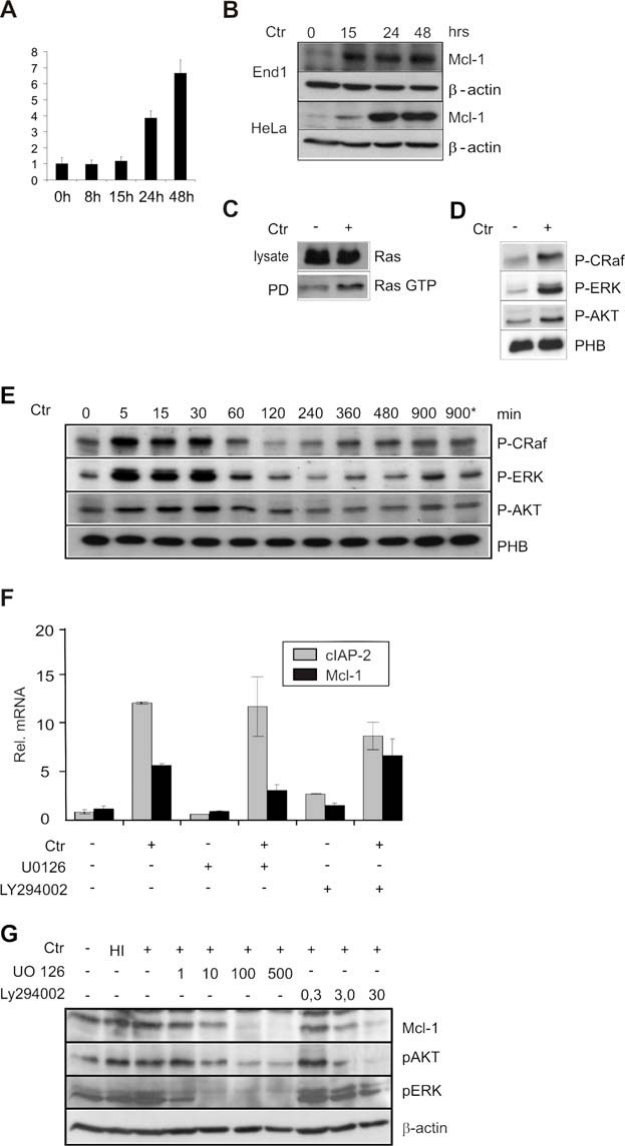
Mcl-1 is up-regulated via MAPK pathways during *C. trachomatis* infection. (A,B) HeLa cells were infected with *C. trachomatis* (MOI 3) and the expression of Mcl-1 was determined in HeLa cells by q-PCR (A) and in HeLa and End-1 cells by immunoblot analysis, at various time points post infection (B). (C) Infection with *C. trachomatis* leads to the activation of Ras in the host cells. Serum-starved HeLa cells were infected with *C. trachomatis* for 15 h and active Ras GTP was pulled down as mentioned in Materials and Methods S1. Total Ras in the lysates and in the pull down sample (PD) was monitored. (D) HeLa cells were infected with *C. trachomatis* (Ctr) at a MOI of 3 for 20 h and the activation of Raf, ERK and AKT was checked by immunoblot analysis using phospho-specific antibodies. Prohibitin (PHB) was used as loading control. (E) Early time points of the experiment described under (D). HeLa cells were infected for the indicated time points (minutes). One control with heat inactivated bacteria (900*) was included. (F) Up-regulation of Mcl-1 mRNA is dependent on MEK-1. Cells were infected either in the presence or absence of 10 µM of U0126 or 62.5 µM of LY294002 for 20 h. The cells were then lysed and the expression of Mcl-1 and cIAP-2 was monitored by q-PCR analysis. Shown are the data from three independent experiments. The error bars represent the ±SD of the mean. (G) MEK-1 and PI3K involved in the regulation of Mcl-1 protein levels. Cells were infected with heat inactivated (HI) or living *C. trachomatis* and the MAPK inhibitors U0126 (1, 10, 100 and 500 µM) and LY294002 (0.3, 3 and 30 µM) were added. The cells were then lysed at 20 h post infection and the protein levels of Mcl-1 were monitored by immunoblot analysis. Actin was used as a loading control.

Cells infected with *C. trachomatis* have previously been demonstrated to resist the activation of Bax and Bak to prevent mitochondrial outer membrane permeabilization and the release of cytochrome *c* from the mitochondria [26]. Using RNA interference screens, we identified host factors which upon knockdown sensitized infected cells for apoptosis induced by ligation of the TNF receptor (unpublished data). Since a dominant role of anti- and pro-apoptotic Bcl-2 family members in regulating mitochondrial outer membrane permeabilization is well documented, we searched for such factors among the hit list. The only anti-apoptotic Bcl-2 family member identified by this approach was Mcl-1. In addition, Mcl-1 has previously been demonstrated to be significantly up-regulated in *C. trachomatis*-infected cells [27], [28]. To confirm the latter observations, we tested for the mRNA and protein levels of Mcl-1 in *C. trachomatis*-infected cells. Q-PCR and immunoblot analysis revealed that Mcl-1 is strongly up-regulated in a time dependent manner in HeLa cells infected with *C. trachomatis* (Fig. 1A,B). Mcl-1 was also up-regulated in infected human primary End-1 cells (endocervical epithelial cells) (Fig. 1B), and HEp-2 cells (data not shown), suggesting that Mcl-1 up-regulation is a general effect of *C. trachomatis* infection. As Mcl-1 has been shown to be profoundly regulated by MAPK signaling, we investigated the role of MAPK pathways towards the up-regulation of Mcl-1 and resistance to apoptosis in infected cells. To perform these experiments, we first checked if the RAS/MAPK cascade is activated in *C. trachomatis* infected cells. Previous studies have also demonstrated that acute infection with *C. trachomatis* can activate MAPK in HeLa cells [29]. Consistent with the published observations, Ras-GTP could be pulled down from the infected cells at 15 h post infection (Fig. 1C) and Raf, ERK and AKT were activated as revealed by their active-phosphorylation status during the early and late phase of infection (Fig. 1D,E). To further analyze if MAPK pathways play any role in modulating Mcl-1 levels in the infected cells, we exploited the use of chemical inhibitors. HeLa cells were infected in the presence or absence of MEK-1 inhibitor U0126 or the PI3K inhibitor LY294002. While treatment of infected cells with U0126 did not exert any significant alteration in the chlamydial growth or cell survival, treatment with LY294002 caused a defect in the fusion of inclusions suggesting that PI3K pathway may play a crucial role in the fusion of chlamydial inclusions (data not shown). Q-PCR analysis of Mcl-1 mRNA levels revealed that Mcl-1 was up-regulated in the infected cells in a MEK-dependent fashion, while cIAP-2 levels remained unaltered (Fig. 1F). As Mcl-1 protein levels were also shown to be influenced by PI3K activation, we infected HeLa cells and the protein levels of Mcl-1 were monitored after treatment with MAPK inhibitors. Inhibition of PI3K leads to complete loss of Mcl-1 protein levels in the control and infected cells (Fig. 1G), suggesting a role for MEK-1 and PI3K pathways in modulating the expression and stabilization of Mcl-1.

### Mcl-1 is required to prevent apoptosis induced by intrinsic and extrinsic pathways

**Figure 2:**
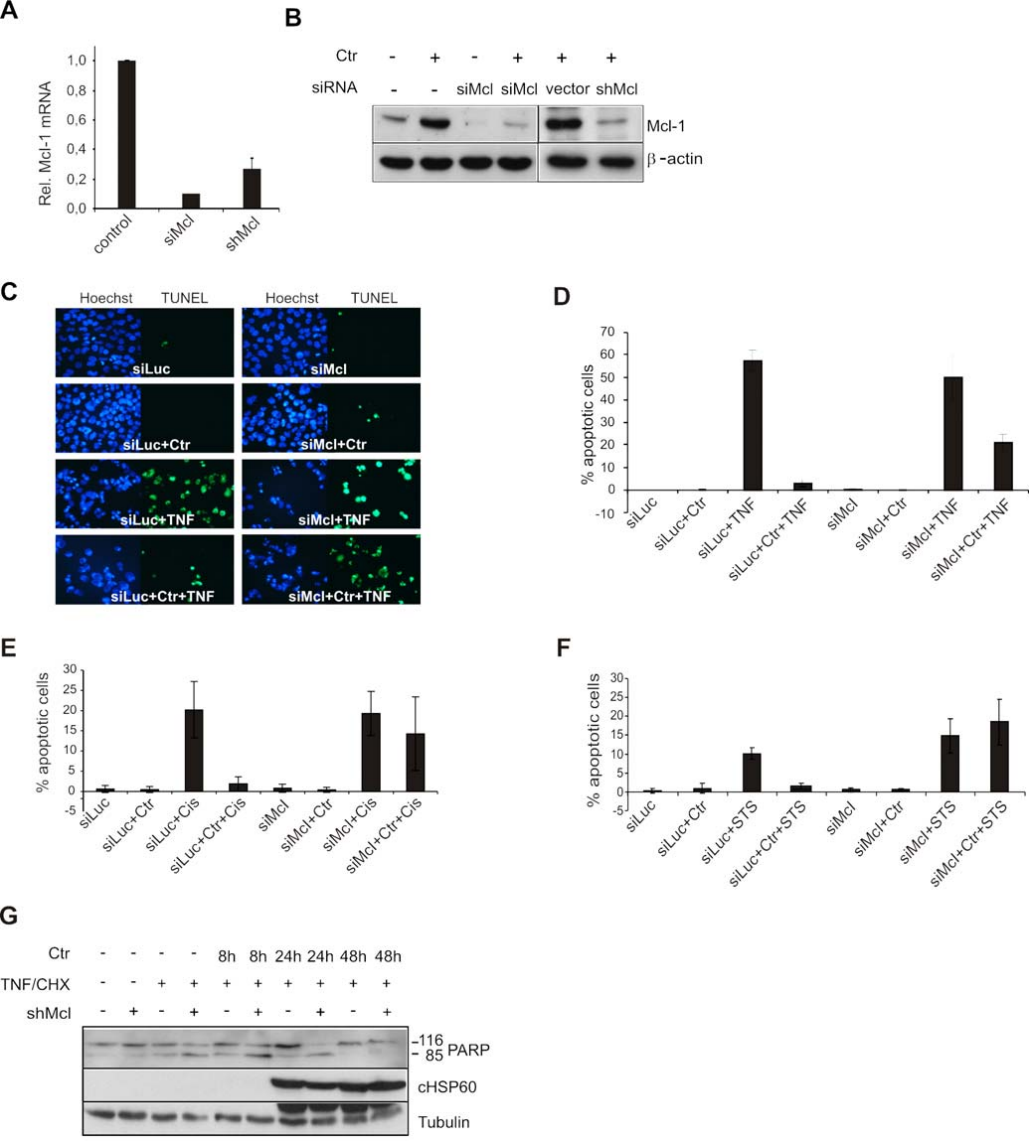
Mcl-1 is required to resist apoptosis induced by various inducers. (A,B) Si- or shRNAs directed against luciferase (siLuc)/empty vector (vector) and Mcl-1 (si/shMcl) were transfected into HeLa cells. The efficiency of silencing Mcl-1 was monitored by q-PCR (A) and by immunoblot analysis (B). (C–F) Cells were then infected (Ctr) at an MOI of 3 and induced to apoptosis by the treatment with TNF/CHX (TNF; C,D), cisplatin (cis; E) or staurosporine (STS; F) as indicated. Cells were fixed, the nuclei were stained with Hoechst (blue) and apoptotic cells were identified by the TUNEL assay (green). For quantification, TUNEL positive cells from each sample were counted from five different fields. Shown are the data from three independent experiments. The bars and the error bars represent the mean±SD, respectively. (G) Sensitization to TNF/CHX mediated cell death by silencing Mcl-1 in infected cells, is confined to the early stages of infection. Control and shMcl-expressing cells were infected as mentioned above and induced to apoptosis with TNF/CHX at various time points of infection. The cells were lysed in sample buffer and the processing of PARP was monitored by immunoblot analysis. Chlamydial Hsp60 (cHSP60) was detected to determine the infection load and Tubulin was used as a loading control.

As *C. trachomatis* infection predominantly blocks apoptosis upstream of mitochondrial outer membrane permeabilization and cytochrome *c* release, we checked if interfering with Mcl-1 expression could sensitize *Chlamydia* infected cells to apoptosis. Transfection of siRNAs against Mcl-1 downregulated Mcl-1 mRNA and protein levels as was analyzed by quantitative realtime PCR (q-PCR) and immunoblot analysis (Fig. 2A,B). As expected, silencing of Mcl-1 using siRNAs sensitized *C. trachomatis*-infected cells to TNF/CHX-induced apoptosis (Fig. 2C,D). As *C. trachomatis* infection can also resist the intrinsic pathway of apoptosis induction [30], the effect of Mcl-1 silencing on cisplatin- and staurosporine-mediated apoptosis was tested in infected cells. Interestingly, silencing of Mcl-1 sensitized infected cells to both these inducers of apoptosis (Fig. 2E–F), confirming that Mcl-1 is primarily required for apoptosis resistance in *C. trachomatis* infected cells. However, sensitization to apoptosis was confined only to cells that carry small inclusions in the range of 4 to 8 µm, while the cells which carry inclusions larger than 10 µm still resisted apoptosis despite the suppression of Mcl-1 (Fig. S1), suggesting that apoptosis resistance depends on the host cell signaling machinery only during the early stages of infection. We therefore performed time course experiments and tested for cleaved PARP as readout for apoptosis to further substantiate our initial finding with a different assay at different time points. Infected HeLa cells were protected from TNF/CHX-induced apoptosis at 24 and 48 h. Depletion of Mcl-1 sensitized cells infected for 24 h whereas those infected for 48 h remained resistant for TNF/CHX comparable to the treated and infected wildtype cells (Fig. 2G). These data demonstrated an infection cycle dependent role of Mcl-1 in the apoptosis resistance of infected cells.

The results were further confirmed in primary End-1cells. Since these cells were not accessible for siRNA transfection (not shown), Mcl-1 protein levels were reduced by treatment with the PI3K inhibitor LY294002. Under these conditions, *Chlamydia*-infected END-1 cells were sensitized to staurosporine-mediated apoptosis (Fig. S2A), suggesting that the observed effect is not confined to transformed cells.

### RAS/MAPK-Mcl-1 axis is required to resist Granzyme B-mediated apoptosis in the infected cells

**Figure 3:**
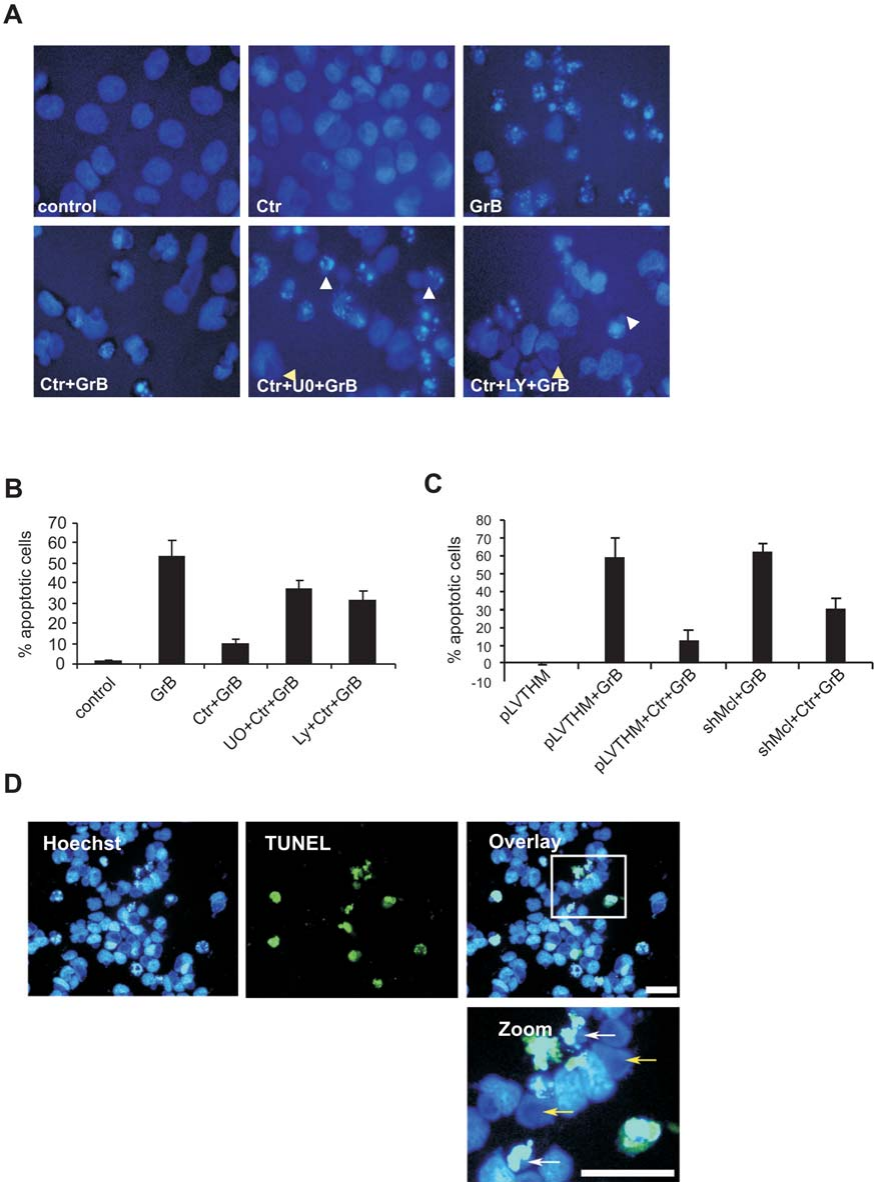
MAPK activation by *C. trachomatis* is required to resist Granzyme B-mediated cell death. (A) Control and infected cells (Ctr) were treated with MAPK inhibitors U0126 (U0) and LY294002 (Ly) 7 h post infection and at 20 h post infection, the cells were induced to apoptosis with GrB/LV for 4 h. The cells were stained with Hoechst to visualize the apoptotic cells with fragmented chromatin. (B) Quantification of the experiment shown in (A). Apoptotic and non-apoptotic cells were counted from five different fields. The bars represent the mean of three independent experiments ±SD. (C) Control cells (pLVTHM) or Mcl-1-depleted cells (shMcl-1) were infected (Ctr) and induced to apoptosis by the treatment with Granzyme B (GrB). (D) Sensitization to GrB-mediated cell death is confined only to cells that carry small inclusions. The cells with an inclusion size of 8–10 µM failed to get sensitized for apoptosis despite the inhibition of MAPKs. Yellow arrows point the big inclusions and white arrows point the small inclusions which fail to resist apoptosis. The white bars represent a length of 50 µM.

Cytotoxic T lymphocyte (CTL)-induced death triggered by the granule exocytosis pathway involves the perforin-dependent delivery of granzymes to the target cell. Previous studies have revealed that *C. trachomatis* infection can resist granzyme B-mediated apoptosis by blocking cytochrome *c* release and caspase activation [5]. Treatment of HeLa cells with 1 µg of GrB/LV induced apoptosis efficiently as revealed by fragmentation of chromatin (Fig. 3A). As expected, cells infected with *C. trachomatis* resisted GrB/LV-mediated cell death and pretreatment of cells with MAPK inhibitors sensitized infected cells to killing by GrB/LV (Fig. 3A,B). In addition, we have established permanent shRNA-mediated silencing of Mcl-1 expression. The efficiency of knock down was validated by realtime PCR and immunoblot analysis (Fig. 2A,B). Control and Mcl-1-silenced HeLa cells were infected and treated with GrB/LV. In line with a role of Mcl-1 in conferring resistance to GrB-induced apoptosis, infected cells depleted of Mcl-1 were strongly sensitized for apoptosis (Fig. 3C). Consistent with our previous observations, sensitization to apoptosis was confined only to cells carrying smaller inclusions (Fig. 3D). The difference in the susceptibility for GrB-induced apoptosis of cells with small and large inclusions did not depend on the shMcl cell line. HeLa cells induced at the late phase of infection, when the cells contained mainly large inclusions, failed to respond to GrB upon inhibition of MAPK (Fig. S2B,C), suggesting that apoptosis resistance during the late stage of infection is independent of MAPK and Mcl-1.

### BH3-only proteins are not degraded during *C. trachomatis* infection

**Figure 4:**
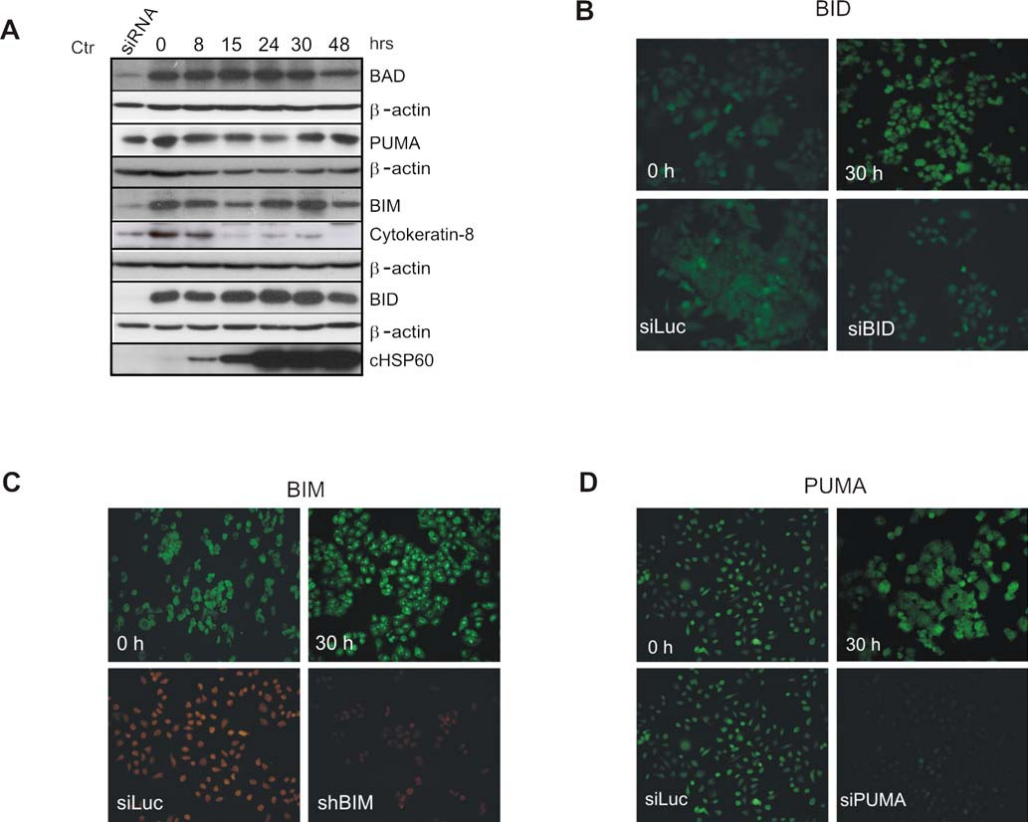
BH3-only proteins are not degraded during *C. trachomatis* infection. (A) HeLa cells were either transfected with the siRNA (siRNA) to downregulate the respective BH3-only protein or infected with *C. trachomatis* (Ctr) (MOI 3) for the indicated time points (h). Infected cells were immediately lysed with sample buffer as described in experimental procedures. The proteins were separated by SDS PAGE and immunoblots are performed to detect the protein levels of Bad, Bim, Bid and PUMA. Bacterial Hsp60 (cHSP60) was used as an infection marker and β-actin as a loading control. Activity of *chlamydial* protease- like activity factor, CPAF, was checked by detecting the levels of keratin-8. Immunofluorescence analysis of cells infected with *C. trachomatis* for expression of (B) BID, (C) BIM and (D) PUMA at 0 and 30 h post infection. The specificity of the antibody was verified by staining cells transfected with validated si- or shRNAs directed against control (siLuc) and the respective genes.

Previous studies have demonstrated that mitochondrial outer membrane permeabilization in cells infected with *C. trachomatis* is primarily blocked by the degradation of the BH3-only proteins in a proteasome-dependent manner [21]–[23]. To test if BH3-only proteins are degraded during *C. trachomatis* infection under our experimental settings, we checked for the protein levels of Bim, Bad, Puma and Bid by immunofluorescence as well as by immunoblot analysis (Fig. 4, S3, S4). HeLa cells were infected with *C. trachomatis* and cells were lysed directly in sample buffer at various time points post infection as mentioned in the methods. As seen in figure 4A, the protein levels of Bad, Bim, PUMA and BID almost remained constant during the infection time course. Quantification of the bands was performed by densitometric analysis (see Materials and Methods S1) and the results were plotted (Fig. S5). Antibody specificity was once more demonstrated by RNAi and immunoblot analysis of the respective genes (Fig. 4A). Besides the BH3-only proteins, keratin 8 has been reported to be cleaved in cells infected with *C. trachomatis* [31]. We therefore tested the same samples for keratin 8 cleavage to rule out major differences between our infection conditions and the published ones. As shown in figure 4A, keratin 8 levels strongly decreased with the onset of chlamydial growth indicated by increasing cHSP60 levels, confirming keratin 8 as a substrate in these infected cells. In parallel, HeLa cells grown on coverslips and infected with *C. trachomatis* were fixed at various time points post infection and stained for Bim, Bad, Bid or Puma with the respective antibodies (for details see Materials and Methods S1). As shown in the figure S4, despite the presence of chlamydial inclusions, the intensity of the fluorescence did not decrease during the infection time course, which implies that there is no degradation of these proteins. To check if the antibodies were specific for the proteins of interest, siRNAs were used to silence the respective genes, and the transfected cells were used as a negative control for the immunofluorescence studies (Fig. 4B–D). In summary, these data suggested that degradation of BH3-only proteins cannot account for the apoptosis resistance in our infection protocol.

### Loss of Mcl-1 can rescue mitochondrial outer membrane permeabilization and caspase activation in the infected cells upon apoptosis induction

**Figure 5:**
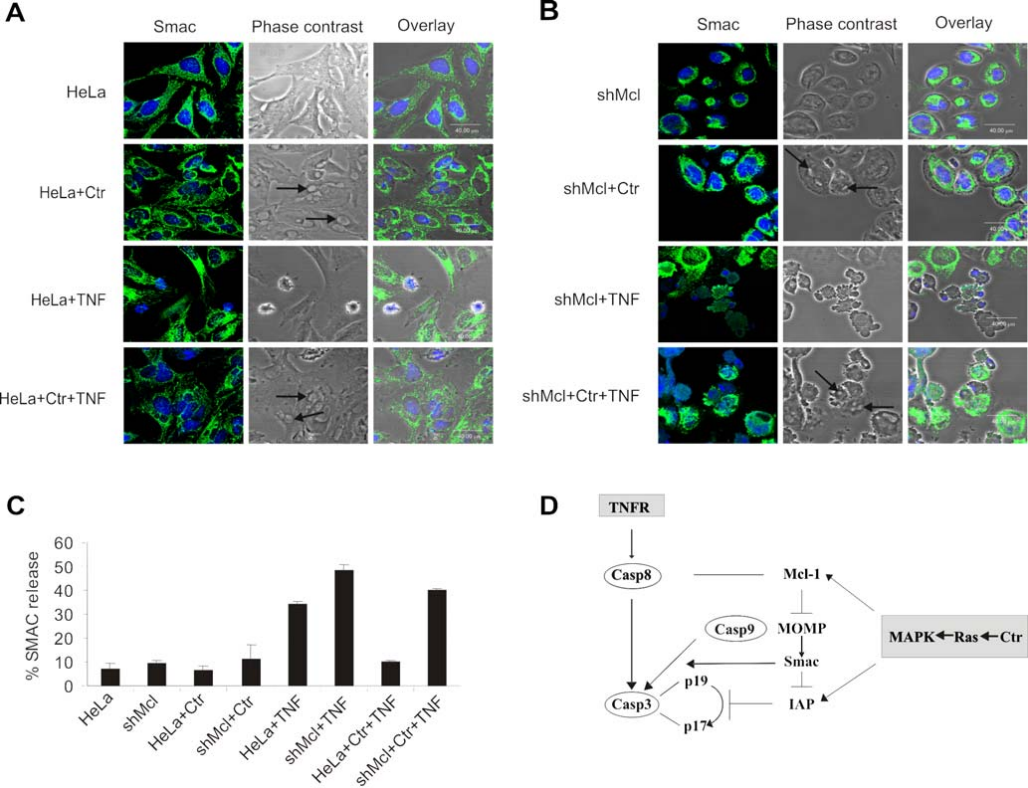
Mcl-1 is primarily required to resist mitochondrial outer membrane permeabilization and caspases activation in *C. trachomatis* infected cells. (A,B) Mcl-1 is required for preventing the release of Smac from the mitochondria of *C. trachomatis* infected cells upon TNF/CHX induction. Control (HeLa cells with empty vector) (A) and shMcl cells (B) were infected at an MOI of 5 and treated with TNF/CHX as mentioned in Experimental procedures. Shown are images from one representative experiment. Smac was stained in green and nuclei in blue. The arrows point to chlamydial inclusions (C) Quantification of cells with released Smac (for details see Experimental procedures). Shown are the data from three independent experiments. Error bars represent the ±SD of the mean. (D) Model of signaling cascades involved in apoptosis inhibition during the early phase of *Chlamydia* infection. Infection induces the activation of MAPK pathways resulting in the upregulation and stabilization of Mcl-1 and IAP. Mcl-1 upregulation prevents the mitochondrial outer membrane permeabilization and the release of the IAP inhibitor Smac/DIABLO. IAPs prevent the activation of caspases, particularly the conversion of the caspases-3 inactive p19 fragment to the active p17 fragment.

We have previously demonstrated that caspase activation upon TNF/CHX-mediated apoptosis induction in *C. trachomatis*-infected cells could be blocked by the up-regulation of cIAP-2 and by the stabilization of IAP-IAP complexes [25]. As MAPK pathway may also influence the stability of IAPs in the infected cells, we have checked for the influence of MAPK pathway on the stabilization of IAPs. Treatment of infected cells with both, U0126 as well as LY294002 caused the destabilization of cIAP-2 protein without altering the mRNA levels in the infected cells (Fig. 1F, S6), underlining the central role of MAPK in the control of anti-apoptotic proteins in *Chlamydia*-infected cells. The finding that MAPK regulate both Mcl-1 and cIAP-2, explained why interfering with MAPK function sensitized infected cells for the induction of apoptosis. The question, however, remained, how interference with apoptosis regulators acting upstream and downstream of mitochondria like Mcl-1 and IAPs, respectively, also affected apoptosis resistance of infected cells. To test if silencing of Mcl-1 can rescue mitochondrial outer membrane permeabilization and release of pro-apoptotic proteins from the mitochondria of infected cells, the release of Smac/DIABLO, a direct inhibitor of IAPs, from the mitochondria after apoptosis induction was analyzed. Loss of Mcl-1 led to the release of Smac in the infected cells when induced to apoptosis with TNF/CHX (Fig. 5A,B; for enlarged versions see Fig. S7). Statistical analysis revealed that the effect observed in shMcl-1 cells was highly significant (P≤0.0003; Fig. 5C). In conclusion, Mcl-1 up-regulation protects the infected cells from mitochondrial outer membrane permeabilization, which would otherwise result in the release of Smac/DIABLO, inhibition of IAPs and the activation of caspases (Fig. 5D).

## Discussion

*Chlamydia* employs multiple pathways to interfere with host cell apoptosis induced via death receptors and stress [32]. The underlying mechanism is currently investigated intensively and involves the activation of NFκB [33], upregulation and stabilization of *inhibitor of apoptosis proteins* (IAPs) [25], [33] and the prevention of Bak and Bax activation [20], [21].

We observed an infection cycle-dependent sensitization profile of infected cells for apoptotic stimuli. The initial phase of the infection cycle, characterized by full sensitivity of the host cell for apoptotic stimuli, is followed by a time window from about 16 to 24 hours post infection during which apoptosis resistance depends on the activation of host anti-apoptotic factors like IAPs and Mcl-1. Recently it has been suggested, that apoptosis inhibition by *C. trachomatis* does neither require IAPs nor Mcl-1 in cells derived from genetically modified mice [34]. Apart from the species and cell type specific differences, these discrepancies could also be attributed to the stage of infection or inclusion size. Consistent with these observations, we detected that host cells are fully resistant to apoptotic stimuli during late phase infections despite the loss of IAPs or Mcl-1 (data not shown). The mechanism underlying apoptosis resistance during the late phase of infection is not known; it is, however, tempting to speculate that bacterial factors then directly interfere with the host cells' apoptosis machinery.

It has been suggested that the block in apoptosis signaling depends on the downregulation of BH3-only proteins in cells infected with *Chlamydia* since depletion of several of these activators of the intrinsic apoptosis pathways has been demonstrated [21]–[23]. Since BH3-only proteins have been shown to inactivate BH1-4 proteins like Mcl-1 [35], infection-induced upregulation of Mcl-1 and depletion of BH3-only proteins would nicely fit to a general and multi-level inhibition of apoptosis upstream of mitochondria. In contrast to these reports, we could, however, not detect significant and long-lasting depletion of BH3-only proteins using immunofluorescence and immunoblot techniques with carefully validated antisera. The observed discrepancy could not be attributed to the infection conditions as we have detected a decrease in protein levels of keratin 8, a substrate of *chlamydial* protease-like activity factor, CPAF [31], with the onset of chlamydial growth, in our experiments. Another possibility is the unspecific degradation of proteins in lysates of infected cells (K.R., T.R., unpublished observations). In any case, degradation of BH3-only proteins can not be the (only) reason for apoptosis resistance in infected cells.

Recently, we have demonstrated that cells infected with *C. pneumoniae* [33] and *C. trachomatis* resist TNF-induced apoptosis primarily by the up-regulation of cIAP-2 and the stabilization of IAP-IAP complexes to block the processing and activation of effector caspases [25]. The resistance to TNF/CHX-induced apoptosis at the caspase-3 level suggests that cells infected with *Chlamydia* resist the release of Smac, which is indeed required during TNF/CHX-induced apoptosis to relieve the inhibition on caspases-3 processing and activation [36]. Here we show that the block in the release of Smac from the mitochondria is reverted in the infected cells upon apoptosis induction suggesting that Mcl-1 is probably the prime block upstream of mitochondria modulating the release of Smac in these cells. Of interest in the same lines is also a recent study made by Mimuro et al. [37], which demonstrates the CagA dependent upregulation of Mcl-1 in gastric pits to prevent the replenishment of gastric epithelium and to sustain *H. pylori* infection. Thus activation of MAPK and upregulation of Mcl-1 may be a more general strategy in combating host cell apoptosis for successful infection.

Our data show that MAPK pathways activated by *C. trachomatis* play a crucial role in maintaining apoptosis resistance in the infected cells in response to different apoptosis inducers. Most importantly, we have investigated strategies employed by *C. trachomatis* to resist GrB-mediated apoptosis, the most relevant apoptotic pathway employed by the CTLs to clear intracellular infections. Mitochondria play a crucial role during several pathways of apoptosis induction including the GrB pathway. Viruses and bacteria have evolved strategies to modulate mitochondrial outer membrane permeabilization either by regulating the levels of host Bcl-2 family proteins or by the release of effector proteins to host cell cytosol to directly influence mitochondrial outer membrane permeabilization [38]. Recent studies have revealed the important role of Mcl-1 in modulating mitochondrial outer membrane permeabilization as destabilizing Mcl-1 can induce the release of cytochrome *c* from the mitochondria [39]. Thus, the strong up-regulation and stabilization of Mcl-1 during acute and persistent infections explains the previous observation of a complete block of mitochondrial outer membrane permeabilization in the *C. trachomatis* infected cells.

*C. trachomatis* infection activates Ras which in turn can activate the Raf/MEK/ERK pathway and the PI3K/AKT pathway. We found that active MEK-1 is responsible for the upregulation of Mcl-1 mRNA while an active PI3K pathway is required for the stabilization of Mcl-1 protein levels. Interestingly, we have detected that the Raf/MEK/ERK and the PI3K/AKT pathway are also required for the infection-induced stabilization of cIAP-2 protein (Fig. S6). This may explain why inhibition of either of these MAPK pathways sensitized infected cells to GrB-, stress- and death receptor-mediated induction of apoptosis (Fig. 3 and data not shown). We therefore propose the following model for apoptosis resistance in *C. trachomatis*-infected cells (Fig. 5E): During the early phase of infection, Mcl-1 and IAPs are upregulated and stabilized in a MAPK-dependent manner. Infection-induced upregulation of Mcl-1 prevents the release of the IAP antagonist Smac/DIABLO whereas IAP upregulation directly prevents the activation of caspases-3. In this model, interference with MAPK signaling affects both branches of apoptosis inhibition up- and down-stream of mitochondria (Fig. 5E). Depletion of Mcl-1 sensitizes infected cells for the release of the strong IAP antagonist Smac/DIABLO whereas downregulation of IAPs may allow the activation of caspases in the absence of significant cytosolic levels of Smac/DIABLO.

## Materials and Methods

### Propagation of *Chlamydia*

*C. trachomatis* LGV serovar L2 was propagated in HEp-2 cells and purified as described previously [40]. Shortly, HEp-2 cells infected with *C. trachomatis* for 72 h were harvested with a rubber policeman followed by a low speed centrifugation at 500×g at 4°C for 10 min (Hermle). Cells in the pellet were ruptured using glass beads and the lysates were centrifuged as before. The supernatants were removed and centrifuged at 45,000×g for 45 min at 4°C in a SS34 rotor (Sorvall Instruments) to pellet *Chlamydia*. *Chlamydia* were washed in SPG–buffer (0.22 M Sucrose, 10 mM Na_2_HPO_4_, 3.8 mM KH_2_PO4, 5 mM Glutamate, pH 7.4) and stored at −75°C. Fresh stocks were used for each experiment.

### Cell culture and infection with *Chlamydia trachomatis*

HeLa and HEp-2 cells were cultured in RPMI-1640 medium supplemented with 10% fetal calf serum (Gibco BRL) at 37°C in 5.0% CO_2_. End1 cells were cultured in a medium containing 1∶1 mixture of Dulbecco's modified Eagle's medium and Ham's F12 medium containing 10% fetal bovine serum. HeLa cells were infected in the presence or absence of MAPK inhibitors U0126 (Cell Signaling Technology) at a final concentration of 10 µM, LY 294002 (Calbiochem) at a final concentration of 65 µM. Cells were infected with *C. trachomatis* with an MOI of 3–5 in RPMI with 5% FCS at 35°C for 2.5 h. After the medium was exchanged for fresh medium with 10% FCS, infected cells were incubated for 22 h at 35°C. The cells were then induced to apoptosis with various apoptosis inducers as described below.

### Transfection of siRNAs and subsequent infection

To inhibit expression of genes by siRNAs, 50,000 cells/well were seeded in a 12-well plate at least 20 h prior to transfection. Short interfering RNAs designed for the inhibition of the genes under investigation and for luciferase (siLuc) as negative control were transfected using the Transmessenger transfection kit or the RNAiFect transfection kit (Qiagen). One day post transfection, the nearly confluent cells were infected with *C. trachomatis* and 24 h later the samples were analysed by immunoblot and apoptosis analysis. SiRNAs targeting the following sequences were employed in this study: siLuc- 5′-AACUUACGCUGAGUACUUCGA-3′, siMcl-1 [2] 5′-AAGAAACGCGGUAAUCGGACU-3′, siMcl-1 [3] 5′-AAGGACACACAAAGCCAATGG-3′, siBim 5′-CGGAGACGAGTTTAACGCTTA-3′, siBad 5′-ACGAGTTTGTGGACTCCTTTA-3′, siPuma 5′- CAGCCTGTAAGATACTGTATA-3′, siBid 5′-TAGGGACTATCTATCTTAATA-3′. Note that for silencing of Mcl-1, we have transfected both the siRNAs together at a final concentration of 60 nM each.

### Apoptosis induction

Infected and control cells were induced to apoptosis with cisplatin (Sigma) at a final concentration of 60 µM for 15 h or with 40 ng/ml of human recombinant TNFα (Pharmingen) with 2 µg/ml of cycloheximide (Sigma) for 4 h or with 1.5 µM of staurosporine (Sigma) for 5 h. For induction of apoptosis with GrB, human lymphocytes derived GrB (1 µM) was mixed with lentiviral particles as described before [41] and then added to cells in the presence of polybrene (Sigma). Four hours post induction, the cells were fixed with 3% paraformaldehyde. Note that cisplatin was added at 7 h post infection for 15 h and TNF/CHX, STS and GrB/LV were added around 20 h post infection for 4–5 h.

### TUNEL assays

Apoptotic cells were detected by the DeadEnd™ Fluorometric Terminal dUTP Nick End Labeling (TUNEL) assay according to manufacturer's instructions (Promega). HeLa cells were transfected and infected as described above. One day post transfection cells were trypsinized and 50,000 cells/well were seeded in a 12 well plate on glass coverslips. After apoptosis induction, cells were fixed with 3% PFA for 30 min at room temperature, washed twice with PBS and permeabilized with 0.2% Triton-X-100 in PBS for 10 min. After washing with PBS, cells were covered with 25–35 µl of Equilibration buffer at room temperature for 10 min. Then cells were labeled with fluorescein-12-dUTP for 60 min. The reaction was stopped by addition of 2-fold SSC for 15 min, washed with PBS and mounted on glass slides with Moviole. Quantification was performed by counting TUNEL stained cells from various fields. Approximately 500 cells were counted per sample for statistical analysis.

### Smac release assay

Control and Mcl-1 knockdown cells were grown on coverslips and infected with *C. trachomatis*. 24 h post infection, apoptosis was induced in infected and control cells with TNFα and cycloheximide. After 6 h, the cells were fixed with 4% PFA. The cells were then washed once with PBS and permeabilised with 1% Triton/PBS for 10 min. Blocking was done using 1% BSA and 0.05% Tween 20 in PBS, for 30 min. The samples were incubated overnight with anti Smac antibody (BD Pharmingen) at a dilution of 1∶100, in PBS. The samples were washed twice with PBS and the bound antibodies were detected using Anti-Mouse Cy-2 conjugated secondary antibody. The coverslips was examined by confocal microscopy under a Leica confocal microscope with TCS software in accordance with established methods. Five fields were selected randomly for each sample and digitally recorded. In the untreated cells, Smac has a mitochondrial localization which results in a punctate staining. After mitochondrial outer membrane permeabilization, Smac localises to the cytosol resulting in a strong reduction of punctate and total Smac staining as previously described [42]. For each field, the latter cells were counted manually and the percentage of such cells in the total number of cells was calculated.

## Supporting Information

Materials and Methods S1 (0.04 MB DOC)

Figure S1 Sensitization to apoptosis is confined only to cells carrying small inclusions. HeLa cells were transfected with siRNAs directed against Mcl-1, infected with C. trachomatis for 24 h and induced to apoptosis with TNF/CHX as mentioned in Experimental procedures. The chlamydial inclusions are stained in orange and the nuclei are stained in blue with Hoechst dye and fragmented DNA was detected by TUNEL staining (Green). The white arrows in the cells depleted of Mcl-1 point to chlamydial inclusions which fail to resist apoptosis.
(2.81 MB TIF)

Figure S2 (A) Treatment with LY294002 sensitizes C. trachomatis infected End-1 cells to staurosporine-induced apoptosis. End-1 cells were infected with C. trachomatis at an MOI of 3 with or without the presence of 10 µM of LY294002. The cells were treated with staurosporine at 24 h post infection. The cells were lysed in sample buffer and the protein levels of cleaved PARP, Mcl-1, pAKT were detected by immunoblot analysis. Actin was used as a loading control and the extent of infection was monitored by checking the Chlamydia Hsp60 levels. (B,C). Cells carrying large inclusions (>8 µm) are not sensitized to GrB/LV-mediated apoptosis despite the inhibition of MAPKs. HeLa cells were infected in the presence of MAPK inhibitors at an MOI of 5 for 24 h and induced to apoptosis with GrB/LV. The cells were fixed and stained for Hoechst 3342 to detect the chromatin. Shown are the data from three independent experiments. The bars and error bars represent the mean+/−SD.
(0.73 MB TIF)

Figure S3 BH3 only proteins are not degraded during C. trachomatis infection. HeLa cells infected with C. trachomatis for various time points were fixed and stained with antisera directed against BAD (A), BID (B), BIM (C) and PUMA (D). Shown are the images obtained from one representative experiment under 20× magnification under an immunofluorescence microscope.
(4.38 MB TIF)

Figure S4 HeLa cells were infected for 30 h and the expression of BIM, BID, BAD and PUMA was checked by immunofluorescence analysis. Shown are the images from one representative field (20×). The Overlay of the green and phase contrast images revealed that despite the presence of Chlamydial inclusions, there is no alteration in the expression levels of these proteins.
(6.34 MB TIF)

Figure S5 Quantification of immunoblots shown in Figure 4A. The immunoblots of BID (A), BAD(B), BIM (C) and PUMA (D) were quantified as described in the supporting methods. Shown are the data from one representative experiment.
(0.31 MB TIF)

Figure S6 MEK-1 and PI3K involved in the regulation of cIAP-2 protein levels. Cells were infected with C. trachomatis and the MAPK inhibitors U0126 (10 and 100 µM) and LY294002 (31, 62, 125 µM) were added. The cells were then lysed at 20 h post infection and the protein levels of cIAP-2, active AKT and ERK were monitored by immunoblot analysis. Prohibitin was used as a loading control.
(0.18 MB TIF)

Figure S7 Enlarged presentation of the Smac immunofluorenscence images shown in figures 5A and 5B.
(6.51 MB TIF)
