# Supplementary material for: Mcl-1 Is a Key Regulator of Apoptosis Resistance in Chlamydia trachomatis-Infected Cells
Source: PLoS One. 2008 Sep 1;3(9):e3102. doi: 10.1371/journal.pone.0003102 (PMC2518856; doi:10.1371/journal.pone.0003102)
Supplement: Materials and Methods S1 — (0.04 MB DOC) [file pone.0003102.s001.doc]

###### Supplementary information

**Materials and Methods S1**

Immunofluorescence microscopy

# siRNA transfected cells were seeded on glass coverslips at 48 h post transfection and fixed with 4% PFA/PBS at 60 h post transfection. The fixed cells were washed once with PBS and permeabilised with 1% Triton/PBS for 1 min and blocked (1% BSA, 5% NGS, 0.05% Tween 20 in PBS for 1 h. Nuclei were stained with Hoechst 33342 (Sigma) at a final concentration of 10 µM. The cells with fragmented chromatin were then quantified under a fluorescence microscope from five different fields. For time course experiments, cells were fixed with 4% PFA/PBS after 0, 8, 15, 24, and 48 hours post infection. The samples were incubated with antibodies against the BH3-only proteins Bim (Cell Signaling, mouse Ab #2819), Bid (Santa Cruz biotechnology, rabbit polyclonal, #sc-11423), Puma (Cell Signaling, mouse Ab, #4976), and Bad (Santa Cruz biotechnology, rabbit polyclonal, #sc-953), for 1 h. The samples were washed twice with PBS and the bound antibodies were detected using the respective secondary antibodies. Anti-Mouse cy-2 conjugated for Puma and Bim and Anti-Rabbit cy-2 conjugated antibody for Bad and Bim; for the shRNA knockdown cell lines with GFP overexpression, cy-5 conjugated antibody was used. The immunofluorescence microscopy was done using exactly the same conditions for the infected cell samples and the negative control samples with gene silencing.

## Validation of mRNA levels by quantitative realtime PCR

#### siRNA validation was performed as described before [1]. Briefly, 3,000 cells/well were seeded in a 96-well plate one day prior to transfection. siRNAs were transfected in triplicates at a final concentration of 56 nM with 0.5 µl RNAiFect per well using the BioRobot® 8000 system (Qiagen). Knockdown measurements were performed three times independently. After 48 h RNA was isolated using the RNeasy® 96 BioRobot® 8000 system (Qiagen). The relative amount of target mRNA was determined by q-PCR using Quantitect^TM^ SYBR® Green RT-PCR Kit following manufacturer’s instructions (Qiagen). The relative expression levels of target mRNAs were normalized against control transfected cells. GAPDH was used as an internal standard.

Generation of cell lines expressing Mcl-1 shRNAs

Stably shMcl-1 expressing HeLa cells were generated according to [http://www.tronolab.com](http://www.tronolab.com/). Briefly, 293T cells were transfected by calcium phosphate method with the target vectors pLVTH-M-shMcl and viral packaging vectors. After 48 h, lentiviral supernatants were filtered through a 0.45 μm filter (concentrated by ultracentrifugation at 25,000 rpm) and infections of HeLa cells were performed in the presence of polybrene (Sigma). 8 h post infection medium was exchanged and after 48 h GFP-positive cells were enriched by FACS sorting (FACSDiva, Becton Dickinson). Single clones of GFP-positive cells were lysed in sample buffer and the efficiency of gene silencing was checked by quantitative realtime PCR and Westernblot analysis as mentioned above. As control, HeLa cells were transduced with the empty vector and all cell lines were tested for interferon response to exclude non-specific effects. The sequence targeted by the mature shMcl RNA in the Mcl-1 mRNA was: 5’-UAGAGUGUAUACAGAACGAAU-3’.

# SDS-PAGE and immunoblot

For SDS-PAGE, cells were lysed in single detergent buffer (20 mM Tris-HCl pH 7.5, 150 mM NaCl, 1 % NP-40, 1 µgml^-1^ Aprotinin, 0,5 µgml^-1^ Leupeptin, 1 mM Pefabloc, 10 µM Pepstatin) for 15-20 min on ice and sonicated twice for 15 sec. Lysates were cleared by centrifugation for 10 min at 13,000 rpm, sample buffer [2] was added to these lysates and boiled at 90°C for 2 min before loading onto the gels. Cells infected with *C. trachomatis* were washed once with PBS and directly lysed on ice in sample buffer with β-mercaptoethanol or DTT as a reducing agent. The lysed cells are collected and loaded on to SDS-PAGE after boiling the lysates to 90°C for 5 min. After separation the proteins were transferred to PVDF-membranes. For immunoblot analysis membranes were blocked with 3% BSA in TBS with 0.5 % Tween-20 for 2 h and incubated with antibodies directed against C-Raf-pS338 (Cell Signaling Technology), C-Raf (C-12, Santa Cruz Biotechnology), pan-Ras (Pharmingen), phospho-ERK (E-10 from Cell Signaling Technology), ERK2 (C-12 from Santa Cruz Biotechnology), phospho-AKT (Pharmingen), Bim (Sigma), Puma (Sigma), Bad (Cell Signaling Technology), Bid (Santa Cruz Biotechnology), PARP (Santa Cruz Biotechnology), Prohibitin (Neomarkers), β-actin (Sigma), active caspase-3 (Cell Signaling Technology), cIAP-2 (Pharmingen) or Mcl-1 (Pharmingen). Antigen antibody complexes were detected by horseradish peroxidase coupled antibodies (Pharmingen) followed by enhanced chemiluminescence (NEN).

**Western blot data analysis**

The blots were scanned using a Canon Scan 5000F scanner. Quantification was performed by densitometry analysis using Adobe Photoshop CS3 (Adobe Systems Incorporated). An area around each band was selected and the mean intensity as calculated by the software was noted. The mean intensity of a region on the blot near the bands was taken as a “blank”. The total intensity of any band was then calculated as the difference between mean intensity of the band and the blank, multiplied by the pixel size of the area selected around the band. The total intensities of bands were normalized using the actin control. The values were plotted on a relative scale with a value of 100% assigned to the 0 h value in each graph.

Ras-GTP pulldown

For precipitation of active Ras, HeLa cells were serum starved for 6-8 h and infected with *C. trachomatis* at an MOI of 3. The infected cells were washed once with PBS and active Ras-GTP was pulled down using the EZ-Detect Ras activation Kit (Pierce) following manufacturer’s instructions.

**References S1**

1. Machuy N, Thiede B, Rajalingam K, Dimmler C, Thieck O et al. (2005) A global approach combining proteome analysis and phenotypic screening with RNA interference yields novel apoptosis regulators. Mol Cell Proteomics 4: 44-55.

2. Laemmli UK (1970) Cleavage of structural proteins during the assembly of the head of bacteriophage T4. Nature 227: 680-685.
