# Supplementary figures and images for: Mcl-1 Is a Key Regulator of Apoptosis Resistance in Chlamydia trachomatis-Infected Cells
Source: PLoS One. 2008 Sep 1;3(9):e3102. doi: 10.1371/journal.pone.0003102 (PMC2518856; doi:10.1371/journal.pone.0003102)

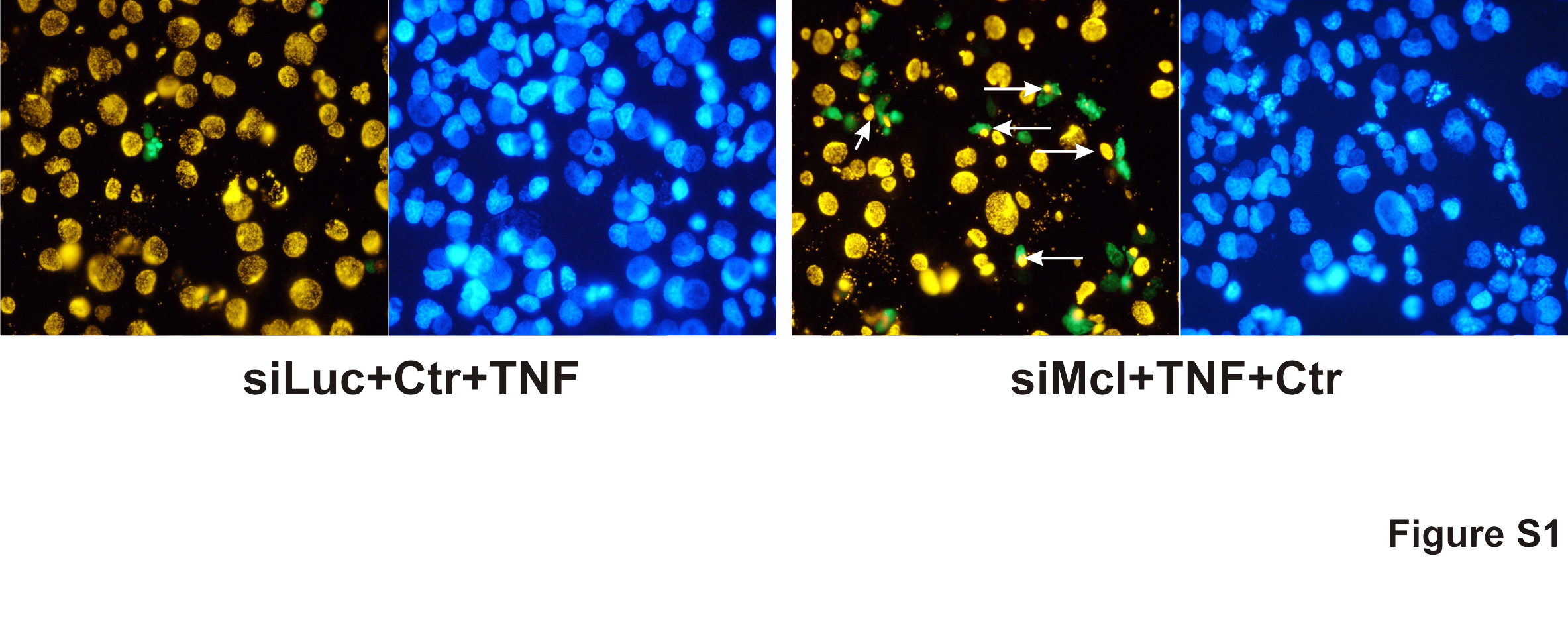

Supplement: Figure S1 — Sensitization to apoptosis is confined only to cells carrying small inclusions. HeLa cells were transfected with siRNAs directed against Mcl-1, infected with C. trachomatis for 24 h and induced to apoptosis with TNF/CHX as mentioned in Experimental procedures. The chlamydial inclusions are stained in orange and the nuclei are stained in blue with Hoechst dye and fragmented DNA was detected by TUNEL staining (Green). The white arrows in the cells depleted of Mcl-1 point to chlamydial inclusions which fail to resist apoptosis. (2.81 MB TIF) [file pone.0003102.s002.tif]

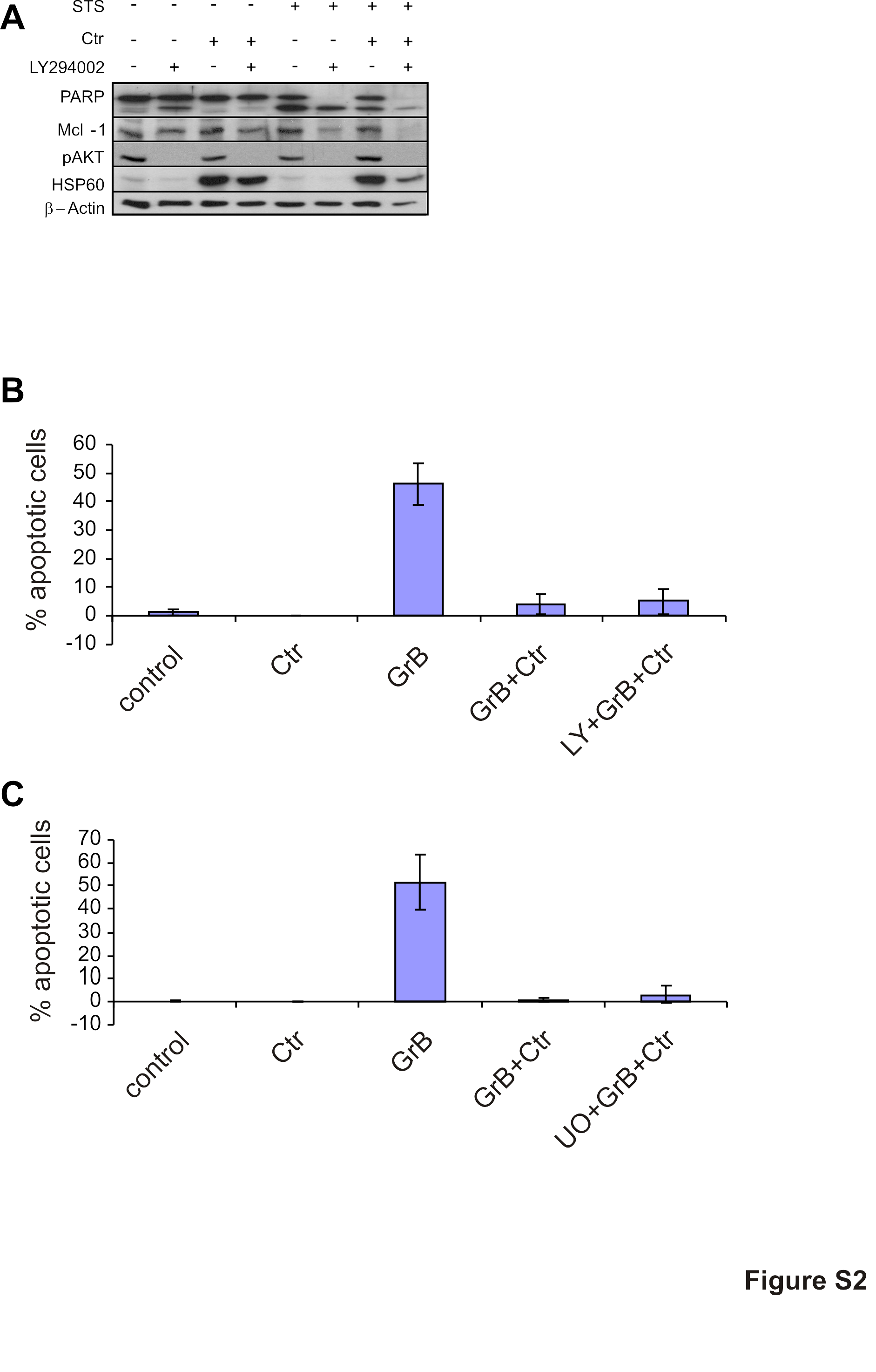

Supplement: Figure S2 — (A) Treatment with LY294002 sensitizes C. trachomatis infected End-1 cells to staurosporine-induced apoptosis. End-1 cells were infected with C. trachomatis at an MOI of 3 with or without the presence of 10 µM of LY294002. The cells were treated with staurosporine at 24 h post infection. The cells were lysed in sample buffer and the protein levels of cleaved PARP, Mcl-1, pAKT were detected by immunoblot analysis. Actin was used as a loading control and the extent of infection was monitored by checking the Chlamydia Hsp60 levels. (B,C). Cells carrying large inclusions (>8 µm) are not sensitized to GrB/LV-mediated apoptosis despite the inhibition of MAPKs. HeLa cells were infected in the presence of MAPK inhibitors at an MOI of 5 for 24 h and induced to apoptosis with GrB/LV. The cells were fixed and stained for Hoechst 3342 to detect the chromatin. Shown are the data from three independent experiments. The bars and error bars represent the mean+/−SD. (0.73 MB TIF) [file pone.0003102.s003.tif]

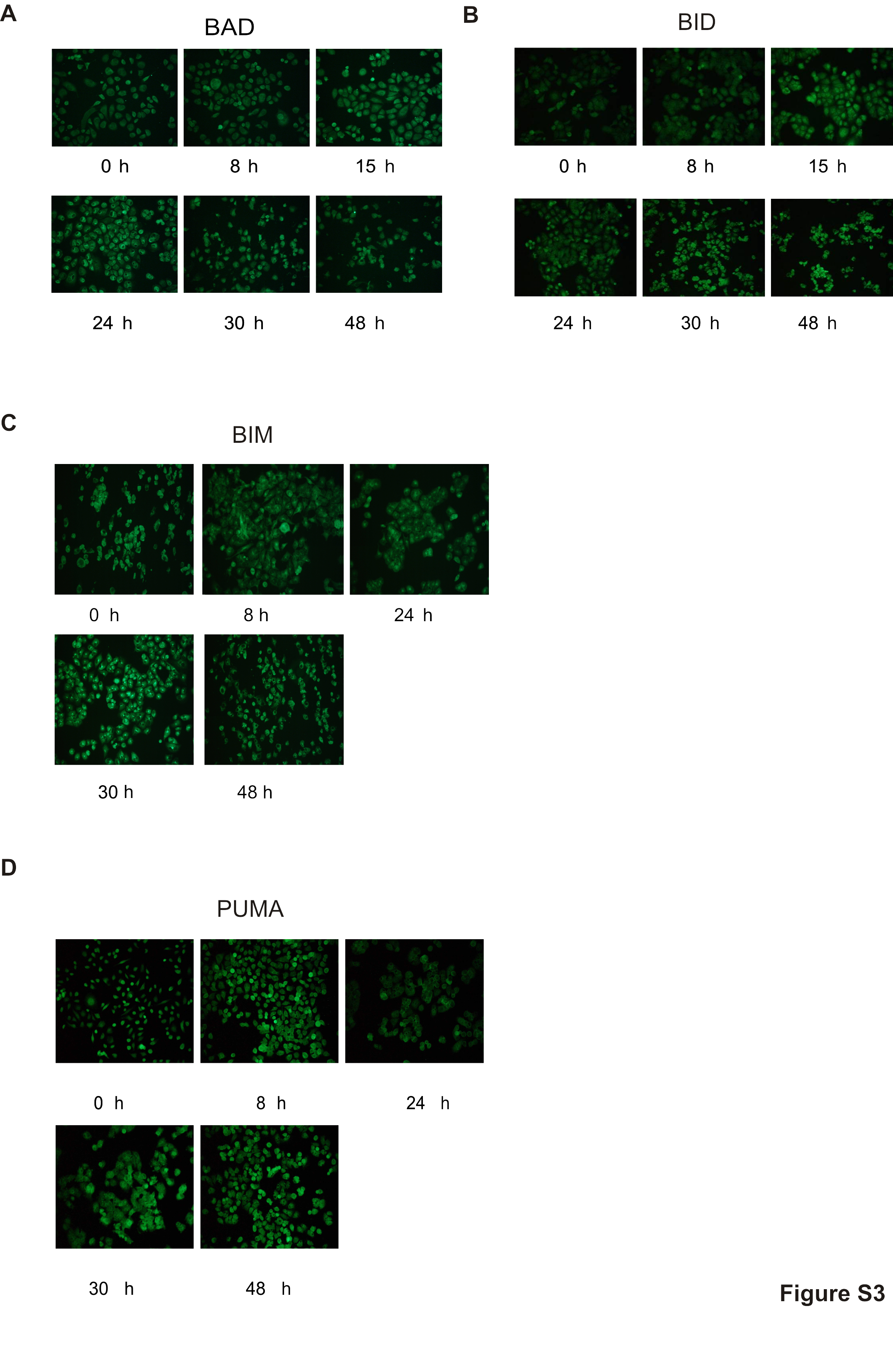

Supplement: Figure S3 — BH3 only proteins are not degraded during C. trachomatis infection. HeLa cells infected with C. trachomatis for various time points were fixed and stained with antisera directed against BAD (A), BID (B), BIM (C) and PUMA (D). Shown are the images obtained from one representative experiment under 20× magnification under an immunofluorescence microscope. (4.38 MB TIF) [file pone.0003102.s004.tif]

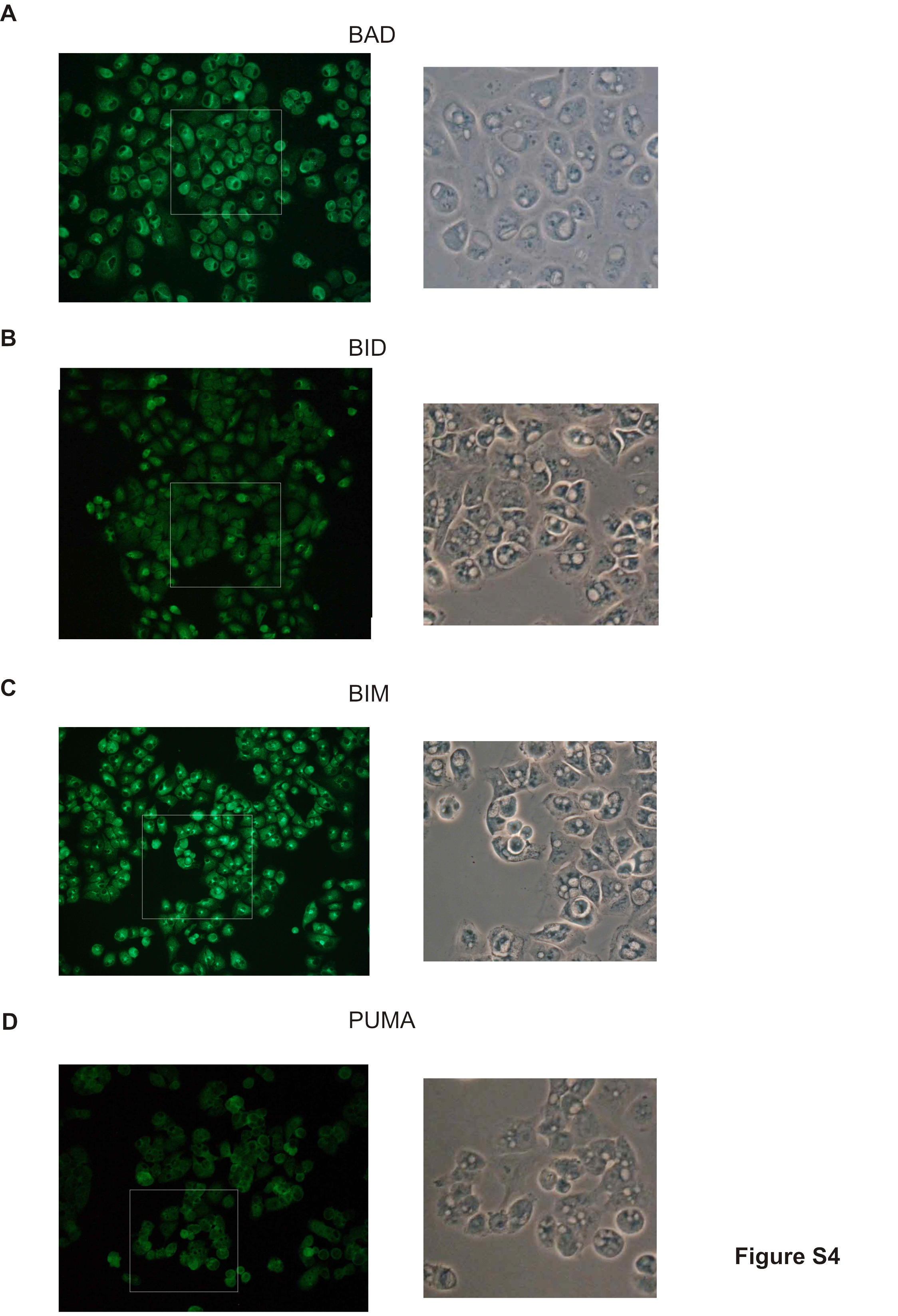

Supplement: Figure S4 — HeLa cells were infected for 30 h and the expression of BIM, BID, BAD and PUMA was checked by immunofluorescence analysis. Shown are the images from one representative field (20×). The Overlay of the green and phase contrast images revealed that despite the presence of Chlamydial inclusions, there is no alteration in the expression levels of these proteins. (6.34 MB TIF) [file pone.0003102.s005.tif]

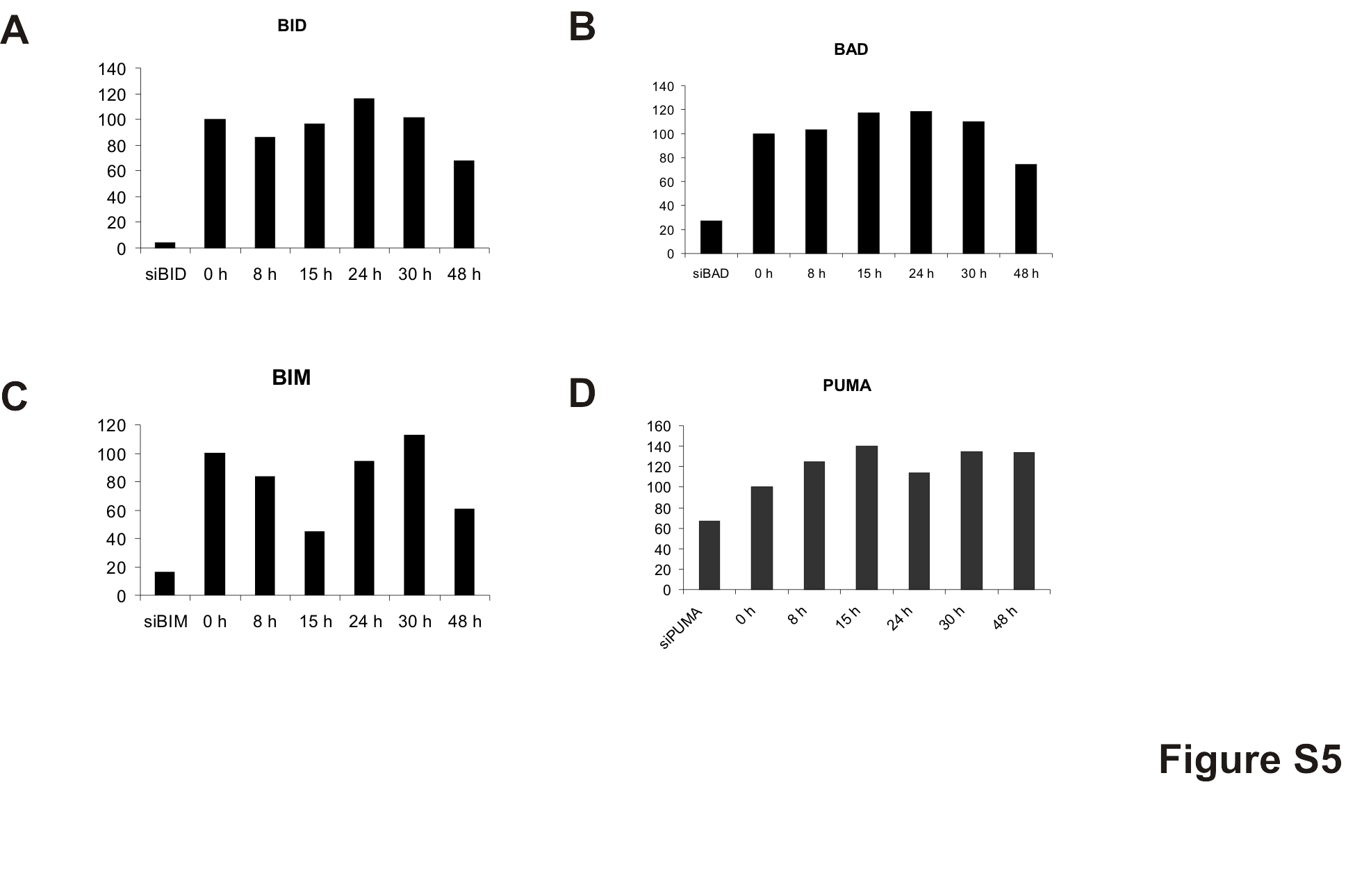

Supplement: Figure S5 — Quantification of immunoblots shown in Figure 4A. The immunoblots of BID (A), BAD(B), BIM (C) and PUMA (D) were quantified as described in the supporting methods. Shown are the data from one representative experiment. (0.31 MB TIF) [file pone.0003102.s006.tif]

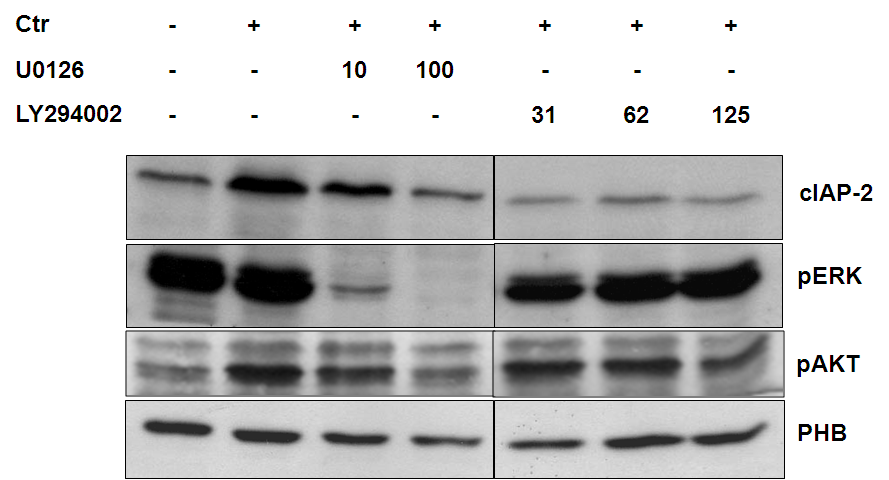

Supplement: Figure S6 — MEK-1 and PI3K involved in the regulation of cIAP-2 protein levels. Cells were infected with C. trachomatis and the MAPK inhibitors U0126 (10 and 100 µM) and LY294002 (31, 62, 125 µM) were added. The cells were then lysed at 20 h post infection and the protein levels of cIAP-2, active AKT and ERK were monitored by immunoblot analysis. Prohibitin was used as a loading control. (0.18 MB TIF) [file pone.0003102.s007.tif]

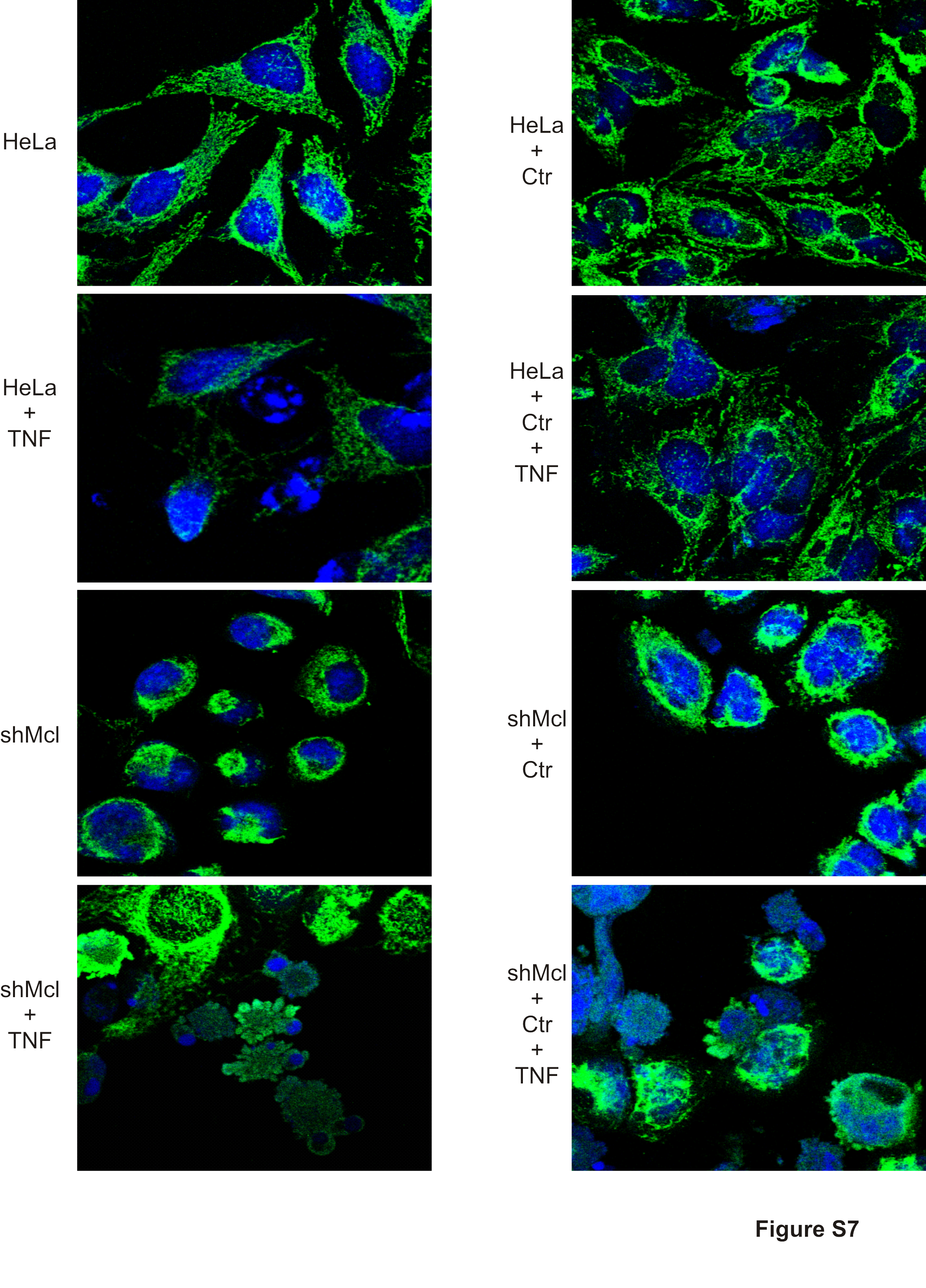

Supplement: Figure S7 — Enlarged presentation of the Smac immunofluorenscence images shown in figures 5A and 5B. (6.51 MB TIF) [file pone.0003102.s008.tif]
